# 

**Published:** 1890-04

**Authors:** 


					﻿JLFR.IL, 1890.
OLD SERIES
Vol. xxv
NEW SERitS
Vol. VII.-No. 2.
1855 4
MWW
Hedu^u
JDDRNAE



W. F. WESTMORELAND, M.D.
H. V. M. MILLER, M.D., LL.D.
VIRGIL 0. HARDON, M.D.
F. W. McRAE, M.D., M’n’g’ng Ed.
/PUBLISHED BY JAMES P.HARRISON&CCK
KsC___________i. sStlanta.ga. ££
SUBSCRIPTION b 12.50 A YEAR.
				

